# Prevalence of high-risk human papillomavirus infections and associated factors among women living with HIV in Shashemene town public health facilities, Southern Ethiopia

**DOI:** 10.1186/s12905-023-02279-2

**Published:** 2023-03-23

**Authors:** Tariku Megersa, Sisay Dango, Kebede Kumsa, Kebebew Lemma, Bikila Lencha

**Affiliations:** 1 Shashemene Comprehensive Specialized Hospital, Shashemene, Oromia Regional State Ethiopia; 2 Department of Public Health, Madda Walabu University Shashemene Campus, Shashemene, Ethiopia

**Keywords:** Human papillomavirus, Women living with HIV, Cervical cancer, Ethiopia

## Abstract

**Background:**

Every year, large number of women are suffering from cervical cancer. Particularly women living with HIV are at high-risk of being suffered with it. Early testing of high-risk human papillomavirus infection can significantly reduce the incidence of cervical cancer. However, lack of early and regular testing has been identified as one of the major problems among risky populations.

**Methods:**

Institutional-based cross-sectional study design was conducted among women living with HIV in Shashemene town public health facilities with a total sample size of 406 from February 1–March 30, 2022. Systematic random sampling technique was employed to select the study subjects. A structured questionnaire and checklist was used to collect data. The collected data were cleaned, coded, and entered into Epi-info version 7.2.5 and exported to statistical package for social science version 24 for analysis. Finally, bi-variable and multivariable logistic regression analyses were performed to identify determinants of high-risk human papillomavirus. Odds ratio with 95% confidence interval was used to test association between exposure and outcome under study and *p*-value < 0.05 was considered significant.

**Results:**

The prevalence of high-risk HPV infections among study participant was 173(35.2%) with 95% CI (30.5%-40.1%). Prevalence of high-risk HPV type 16, 18 and other high-risk HPV types were 62(15.3%), 23(5.7%) and 58(14.3%), respectively. Having history of sexually transmitted infections [AOR = 3.120; 95% CI (1.977–4.923)], Endline CD4 count < 200 cells/mm3 [AOR = 3.072; 95% CI(1.009–9.350)], Endline HIV viral-load ≥ 50 copies/ml [AOR = 3.446; 95% CI(1.368–8.683)] and more than one-lifetime sexual partner [AOR = 2.112; 95% CI(1.297–3.441)] were significantly associated with high-risk HPV infections.

**Conclusion:**

More than one third of women living with HIV had high-risk HPV. Having history of STI, low CD4 count, high viral load and multiple sexual partners were associated with high risk HPV. HIV positive women with these risk factors should be given special consideration in clinical and public health intervention.

## Background

Cervical cancer (CC) is a type of cancer that occurs in the cell of the cervix, the lower part of the uterus that connects to the vagina [1, 2]. The main cause of CC is a persistent infection of the cervix with human papillomavirus (HPV) [3]. There are more than two hundred (200) different types of HPV genotypes predicted to exist. The majority of them do not cause disease and clear up spontaneously. Based on the frequency of detection in CC, HPV genotypes are sub-divided into high-risk HPV (Hr HPV) types (16, 18, 31, 33, 35, 39, 45, 51, 52, 56, 58, 59, 66 and 68) and low-risk (LR) types (6, 11, 42, 43, 44, 54, 61, 70, 72, and 81**)** [4]. Almost all cases of CC are caused by Hr HPV [5]. HPV Types 16 and 18 are the most common type of Hr HPV and together account for about 70% of CC [6].

World Health Organization (WHO) launched a strategy to reduce the incidence of CC [7], which are the primary prevention of Hr HPV(vaccination to females whose ages are 9 to 14 years old) [8, 9], and secondary prevention (early detection of Hr HPV or screening of pre-cancerous lesion) and treating of women with cervical pre-cancer and invasive cervical cancer(ICC) [10]. The Hr HPV test is a screening test for CC, which detects the presence or absence of HPV, the virus that causes CC. Priority is given to women living with HIV for HPV testing at an earlier age starting from the time HIV is detected and to re-screen every two years [11]. Since, Women living with HIV (WLWH) has a more rapid progression of Hr HPV infection to pre-cancer lesions and CC, and also reduced the likelihood of regression of pre-cancer lesions, and higher rates of recurrence following treatment [12].

Cervical cancer is a health crisis impacting women and their families across the world [13]. Globally, every year an estimated 604,000 new cases, which represent 6.5% of all female cancer, are diagnosed with CC [14]. Over 90% of the highest incidence rates of CC occur in sub-Saharan Africa [15]. Globally, around 342,000 women died from the disease every year and almost 90% of them occurred in low and middle-income countries(LMICs) regions of the world like SSA, Central America, and south-central Asia [10, 14]. In Eastern Africa, every year an estimated 54,560 new cases of CC are diagnosed and about 36,497 women lost their life due to this disease [3]. In Ethiopia, CC is the second most common cancer among women with over 7,445 new cases diagnosed and around 5,338 women died from the disease every year [16].

A Meta-analysis conducted in England shows that; women living with HIV were 2.55 times more likely to acquire any Hr HPV. Study shows that, in nine countries with high HIV prevalence including Ethiopia, the proportion of CC attributable to HIV is 40% or higher, whereas it is less than 5% in 122 countries with much lower HIV prevalence [17].

A study conducted in different countries across the world identified several factors; that may contribute to cervical infection with Hr HPV among Women living with HIV(WLWH), this includes Socio-demographic factors like:- age and educational level was found to be independent factors of acquiring Hr HPV [18, 19]. Similarly, behavioral factors like;—alcohol consumption, smoking of cigarettes, and shisha were found to be a significant independent factors for Hr HPV [20–22]. And also reproductive and sexual health factors like:—having multiple sexual partners, oral contraceptive use, sexual debut before the age of 18 years old, history of previous abortion, inconsistent condom use, and ever had a sexually transmitted infection (medically confirmed) (STI) were found to be a significant independent factor for Hr HPV [20, 23–25]. Additionally, Clinical factors like CD4 cell count and current ART were found to be significant independent factors for Hr HPV [26, 27].

Early testing of Hr HPV can reduce the onset of ICC. A study conducted in North America shows that; a woman whose Baseline HPV was negative had a significantly lower cumulative incidence of CC [28]. Lack of early HPV testing and treatment for Women living with HIV (WLWH) has been identified as one of the severe problems among poor populations, especially in countries where resources and services are limited the probability of progress to ICC is high [29]. The topic is timely and has a great contribution for the attainment of sustainable development goals of reducing maternal mortality.

Addressing the current global direction of CC prevention and control is crucial for the reduction of mortality and morbidity of women from cancer. Early testing of Hr HPV for WLWH is important for the improvement of the burden of CC. Identifying the prevalence of Hr HPV may assist the government in allocating resources and in designing interventions to overcome barriers to CC prevention and control. Therefore, this study was aimed at determining the prevalence of Hr HPV and associated factors among WLWH attending ART clinic at Shashemene town public health facility in 2022.

## Methods

### Study setting and population

An institution based cross-sectional study was conducted in Shashemene town public health facilities, Oromia, Southern Ethiopia. Shashemene town is located 250 km away from Addis Ababa the capital city of Ethiopia. The total population of the town is 382,216 (187,286 males and 194,930 female) [30]. The town has 2 governmental hospitals, 1 private hospital, 4 functional public health centers and 1 private health center, of which currently 3 public health facilities are providing cervical cancer screening service such as HPV screening.

All WLWH who come to Shashemene town public health facilities ART clinic were source population. All WLWH who come to Shashemene town public health facilities ART clinic and tested for HPV during study period were our study population, whereas women who had CC screening less two years, women who had hysterectomy done and impossibility of obtaining samples were excluded from the study.

### Sample size determination

Sample size was determined using single population proportion formula with the assumption of 50% proportion of HR HPV among WLWH was used (since so far no similar study was conducted in similar geographical area and among same cultural groups) and 95% confidence level (CL) and 5% of marginal error(d). Finally, by adding 10% of non-response rate the total sample size was estimated to be 422.

$$\mathrm n=\;(\mathrm z2\mathrm p\;(1-\mathrm p))/\mathrm d2$$

$$\mathrm n=\;\mathrm{Sample}\;\mathrm{size}$$

$$\mathrm d=\;\mathrm{the}\;\mathrm{proportion}\;\mathrm{of}\;\mathrm{sampling}\;\mathrm{error}\;\mathrm{between}\;\mathrm{the}\;\mathrm{sample}\;\mathrm{and}\;\mathrm{the}\;\mathrm{population}\;=\;5\%\;(0.05)$$

$$\mathrm z=\;\mathrm{the}\;\mathrm{standard}\;\mathrm{score}\;(\mathrm{critical}\;\mathrm{value})\;\mathrm{corresponding}\;\mathrm{to}\;95\%\;\mathrm{confidence}\;\mathrm{level}\;=\;1.96$$

$$(1.96)2\;\ast\;0.5(1-0.5)\;/\;(0.05)2\;=384$$

### Sampling procedure

**Fig. 1:**
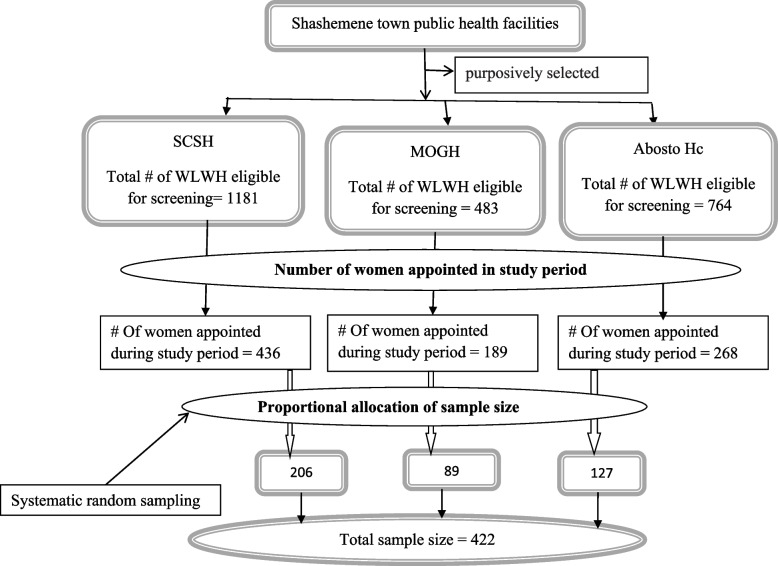
Schematic presentation of sampling technique for the selection of WLWH for Hr HPV screening from Shashemene town public health facilities, Ethiopia, 2022

All public Health facilities currently providing HPV testing in the town namely Shashemene comprehensive specialized Hospital (SCSH), Melka Oda General Hospital (MOGH), and Abosto health center (Hc) were taken. Based on the number of eligible women and women appointed during the study period, the total sample size was allocated proportionally to each health facility. A study subject was selected using systematic random sampling technique. K value was calculated for each health facility and average k value (k = 2) was taken (Fig. 1).

### Data collection tools and procedure

A questionnaire was adapted from different literature's of similar studies [18–24] and adapted to the current aim of the study. The prepared questionnaire included information on socio-demographic characteristics, sexual and reproductive health factors, behavioral factors, and clinical related factors. The questionnaire was prepared in English then translated to local language (Afan Oromo and Amharic) and translated back to English to ensure consistency and accuracy. The data was collected by 6 trained health professionals currently working on CC screening using face-to-face interviews. Patient’s chart was reviewed to retrieve medical information like; CD4 count, viral load, length of being on ART and WHO clinical staging of the patients from patient card after the result is returned.

### Laboratory procedure

A sample of cells for Hr HPV test was collected by a trained health care provider after the patient was placed in a dorso lithotomy position by inserting a swab deep into the cervix and rotating as directed. The health care provider then places the swab in the sleeve. The closed sleeve was kept at room temperature and sent to Shashemene regional laboratory which is found inside Shashemene comprehensive specialized Hospital at ambient temperature in weekly or bi-weekly shipment. At the testing laboratory the sample was kept at 4 °C until extraction (within 3 weeks). DNA extracts are stored temporarily at 4 ºC or for long-term storage, in -80ºC freezer. Then, DNA extraction and high-risk HPV detection from collected cervical and vaginal swabs was conducted. DNA extraction was performed with a modified protocol and the commercial QIA amp kit (Qiagen, Valencia, CA) and the presence of high-risk HPV in the extracted patient DNA was determined with the Cobas 4800 HPV Test. The Cobas 4800 HPV test identifies the presence of HPV type 16, 18 and other Hr HPV (12 genotypes of Hr HPV).

### Data quality management

The quality of data was assured by applying a properly designed and pre-tested questionnaire. The tool was pre-tested on five percent (5%) of the sample size at Dodola General Hospital before the actual data collection to establish its ability to elicit relevant information. Regular supervision and follow-up were made by supervisor. In addition, regular check-up for completeness and consistency of the data was made on daily basis and checking of questionnaire consistency was made. Incomplete questionnaires, was corrected on spot five percent (5%) of known sample was taken from Hawasa regional laboratory and rechecked at Shashemene regional laboratory. regular check-up for proper labeling of collected sample was made. Again in testing unit, expiry date and validity of test kit was regularly checked. For sample tests invalid by the Cobas 4800 HPV test, the DNA from that sample was retested one time to obtain valid results. For repeated invalid result, the final result was recorded as invalid. Improperly labeled sample and invalid result was discarded and considered as non- response rate.

### Variable measurement

Our dependent variable is presence or absence of high risk human papilloma virus. It is a dichotomous variable and labeled as “yes” for High risk HPV and “no” for others (Low risk HPV and not found) as defined under operational definition.

Our independent variables include age (15–24, 25–34, 35–44 and ≥ 44 years), Educational status(illiterate, only reading and writing, primary school, secondary school, and college and above)), marital status (married, widowed and others (single, divorced)), occupation (government employee, house wife, merchants and others (farmer, daily labourer)), ever heard about cervical cancer screening, previous history of CC screening, type of screening (VIA and others (pap smear, HPV screening)), history of chronic disease (hypertension, diabetes), history of STI, years of ART Attendance (< 2 years and ≥ 2 years), baseline and end-line CD4 count (< 200cell/mm^3^ and > 200cell/mm^3^), baseline and end-line HIV viral-load (< 50 copies/ml and ≥ 50 copies/ml), cigarette smoking, drinking alcoholic drinks (beer, wine and alcohols), chewing khat, taking any other drug (other than cigarettes, alcohol or khat), number of birth (≤ 1, and > 1), age at first sexual intercourse (< 18 years and ≥ 18 years), number of life time sexual partner (1 and > 1), number of her husband’s life time sexual partner (1 and > 1), use of modern contraceptive methods and history of abortion.

### Data processing and analysis

The collected data was checked; coded and entered into Epi info version 7.2.5 and exported to SPSS version 24.0 for further analysis. Descriptive statistics like frequency and cross-tabulation were carried out to summarize data. Bivariate logistic regression analysis were carried out to check the association of individual variables with the outcome variable. Those variables with significance level p-value < 0.2 in bivariate analysis was entered into multivariable logistic regression model for further analysis and the backward stepwise (likelihood ratio) were carried out to adjust for the confounding variables. The Hosmer–Lemeshow goodness-of-fit statistic was used to assess whether the model is fit ( p-value 0.396). Odds ratios (OR) and the corresponding 95% confidence intervals (95% CI) were calculated to assess the degree of association between independent and dependent variables. Finally, the variables with a p-value less than 0.05 were considered as statistically significant.

### Operational definition

High risk human papilloma virus (HR HPV): is a type of HPV (HPV type 16, 18, 31, 33, 35, 39, 45, 51, 52, 56, 58, 59, 66 and 68) that can cause cervical cancer and other types of cancer, such as cancers of the anus, vagina, vulva, penis, and oropharynx. Chronic infection with Hr HPV can lead to cell changes that, if not treated, may become cancer [31].

Low risk human papilloma virus (LR HPV):—is a type of HPV (HPV type 6, 11, 42, 43, 44, 54, 61, 70, 72, and 81) that can cause skin warts and respiratory papillomatosis, many of them go away on their own without treatment [31].

## Result

### Socio-demographic characteristics of the women’s

A total of 406 WLWH attending ART clinics in public health facilities of Shashemene town were included in the study making a response rate of 96.3%. Regarding the women’s age majority of women, 198 (48.8%) were in the age group of 35–44 years followed by 173 (42.6%) age group 25–34 years. The mean age of the women was 34 (SD ± 6) years. Regarding the religion of the women’s, majority of the women 182 (44.8%) were followers of the orthodox religion. Regarding the women’s educational status 120 (29.6%) attended primary school and 105(25.9%) were illiterate. Regarding the residence of respondents, the majority of residences 279 (68.7%) were urban dwellers. Regarding the occupation of the women 214 (52.7%) were housewife (Table 1).

**Table 1:** Socio-demographic characteristics of the women living with HIV/AIDS who were screened for cervical cancer, at Shashemene town public health facilities, Ethiopia, 2022

Variables	Category	Frequency	Percent (%)
Age	15–24 years	11	2.7
	25–34 years	173	42.6
	35–44 years	198	44.8
	> = 44 years	24	5.9
Ethnicity	Oromo	177	43.6
	Amhara	130	32
	Wolayita	82	20.2
	Others^a^	17	4.2
Religion	Muslim	114	28.1
	Orthodox	184	44.8
	Protestant	105	25.9
	Catholic	5	1.2
Educational status	Illiterate	105	25.9
	Only reading & writing	63	15.5
	Primary school	120	29.6
	Secondary school	59	14.5
	College & above	59	14.5
Marital status	Married	282	69.5
	Widowed	84	20.7
	Others^b^	40	9.9
Residence	Urban	279	68.7
	Rural	127	31.3
Occupation	Government employee	55	13.6
	House wife	214	52.7
	Merchants	118	29.1
	Other^c^	19	5.2
Husband’s educational level	No formal education	64	15.3
	Primary education	191	47
	Secondary education & above	153	37.7
Husband’ occupation	Farmer	111	27.3
	Merchant	109	28.8
	Driver	45	11.1
	Daily labourer	70	17.2
	Government employee	71	17.5

^a^Kambata, Hadiya Tigre, ^b^single, Divorced, ^c^farmer, daily labourer

### The awareness and clinical factors of the women

Among 406 women’s enrolled in the study, 402(99%), heard about cervical cancer screening. Out of them, 273(67.9%) women got information from health providers. Among study participants, 66 (16.3%) women had cervical cancer screening before this study. Most of the women 174 (57.1%) had a history of STI and 58 (14.3%) women had chronic diseases. Regarding Years of ART attendance, the majority of women, 396 (97.5%) were > 2 years (Table 2).

**Table 2:** Awareness and clinical factors of women living with HIV/AIDS who were screened for cervical cancer, at Shashemene town public health facilities, Ethiopia, 2022

Variables Percent (%)	Value		Frequency
Ever heard about cervical cancer screening (*n* = 406)	Yes	402	99
	No	4	1
First time Source of information	Health professional	273	67.9
	TV/Radio	107	26.6
	Neighbors	22	5.5
Have you had screening	Yes	66	16.3
	No	338	83.3
Type of screening	VIA	54	13.3
	Others^a^	12	2
History of chronic disease^b^	Yes	58	14.3
	No	348	85.7
Have you ever infected with STI?	Yes	174	57.1
	No	232	42.9
Years of ART Attendance	< = 2 Years	10	2.5
	> 2 Years	396	97.5
Previous change of ART regimen	Yes	303	74.6
	No	103	25.4
Baseline CD4 Count (cells/mm^3^)	< 200	50	12.3
	> = 200	356	87.7
End line CD4 Count (cells/mm^3^)	< 200	19	4.7
	> = 200	387	95.3
Baseline HIV Viral load	< 50 copies	274	67.5
	> = 50 copies	132	32.5
Endline HIV Viral load	< 50 copies	375	92.4
	> = 50 copies	31	7.6
WHO clinical stage	Stage 1	404	99.5
	Others^c^	2	0.5

^a^pap smear, HPV screening

^b^hypertension, diabetes

^c^stage 2, 3 & 4

### Behavioral, sexual and reproductive factors

Among 406 women’s enrolled in this study all of them did not have a history of smoking cigarettes and taking any alcoholic drugs. Concerning the history of substance use, 76(18.7%) and 29(7.1%) women used to drink alcoholic (like beer, and wine) and chew khat respectively. Among 406 WLWH enrolled in the study majority, 334 (82.3%) gave more than one birth and 284 (70%) had a sexual debut at/after 18 years old. Concerning lifetime sexual partners, 272 (67%) women’s had an only one-lifetime sexual partners, however, 271(66.7%) of their husbands had more than a one-lifetime sexual partner. About 281 (69.2%) of WLWH used modern contraceptive methods in their lifetime and 305 (75.1%) of women had no history of abortion (Table 3).

**Table 3:** Sexual and reproductive factors of women living with HIV/AIDS who were screened for cervical cancer, at Shashemene town public health facilities, Ethiopia, 2022

Variables	Value	Frequency	Percent(%)
**Behavioral factors**			
Drinking alcohols like beer, wine, alcohol etc	Never	330	81.3
	Yes	76	18.7
Chewing khat	Never	337	92.9
	Yes	29	7.1
**Sexual and reproductive factors**			
Number of birth	> 1	334	82.3
	1	72	17.7
Age at first sexual intercourse	> = 18 Years	284	70
	< 18 Years	122	30
Number of life time sexual partner	1	272	67
	> 1	132	33
Number of her husband’s life time sexual partner	> 1	271	66.7
	1	135	33.3
Use of modern contraceptive methods	Yes	281	69.2
	No	125	30.8
History of abortion	Yes	305	75.1
	No	101	24.9

### Prevalence of high risk human papillomavirus

**Fig. 2:**
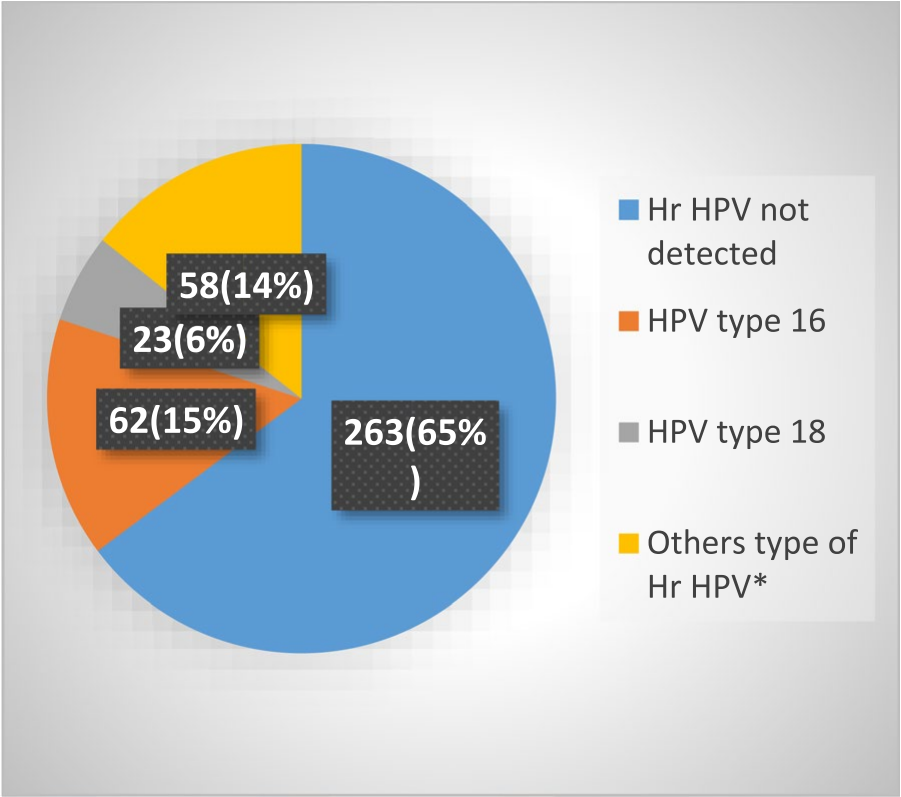
Prevalence of Hr HPV among women living with HIV/AIDS who were screened for cervical cancer, at Shashemene town public health facilities, Ethiopia, 2022 (* = HPV type of Hr HPV, 31, 33, 35, 39, 45, 51, 52, 56, 58, 59, 66, and 68)

The prevalence of Hr HPV among study participants was 143(35.2%) with 95% CI (0.305–0.401). Out of them 62(15.3%) were HPV type 16, 23(5.7%) were HPV type 18 and 58(14.3%) were other Hr HPV was detected (Fig. 2).

### Factor associated with high risk human papillomavirus

On bivariate analysis history of chronic disease, history of STI, baseline CD4 count < 200cells/mm^3^, end line CD4 count < 200cells/mm^3^, baseline HIV viral load >  = 50 copies, endline HIV viral load >  = 50 copies, having early sexual intercourse, having > 1-lifetime sexual partners, and having a husband who have > 1-lifetime sexual partner were identified as predictor of high risk human papillomavirus. On multivariable logistic regression analysis, WLWH who had a history of STI encountered Hr HPV 3-times more than those who did not have, [AOR = 3.120, 95% CI (1.977–4.923)]. Similarly, women who had > 1-lifetime sexual partners were about 2-times higher to be infected with Hr HPV than those who had only one-lifetime sexual partner, [AOR = 2.112, 95% CI (1.297–3.441)]. And also WLWH whose end line CD4 count was < 200 cells/mm3 about 3-times greater to be infected with Hr HPV than women whose endline CD4 count was greater than or equal to 200 cells/mm^3^, [AOR = 3.072, 95% CI (1.009–9.350)]. WLWH whose end line viral load was >  = 50 copies were about 3-times to be infected with Hr HPV than women whose viral load was < 50 copies, [AOR = 3.446, 95% CI (1.368–8.683)] (Table 4).

**Table 4:** Factors associated with Hr HPV among women living with HIV/AIDS at Shashemene public health facilities, Ethiopia, 2022

Variables		Hr HPV		COR	AOR
		Yes	No		
History of chronic disease	Yes	27	31	1.742(0.993–3.056)	1.642(0.873–3.087)
	No	116	232	1	
History of STI	Yes	90	84	3.619(2.361–5.546)	3.120(1.977–4.923)
	No	53	179	1	
Baseline CD4 + count	< 200	24	26	1.838(1.012–3.339)	1.378(0.657–2.889)
	> 200	119	237	1	
Edline CD4 + count	< 200	10	4	3.350(1.288–8.711)	3.072(1.009–9.350)
	> 200	133	259	1	
Baseline HIV viral load	< 50 copies	88	186	1	
	> 50 copies	52	72	1.439(0.937–2.211)	0.899(0.533–1.516)
End line HIV viral load	< 50 copies	122	253	1	
	> 50 copies	21	10	4.355(1.990–9.533)	3.446(1.368–8.683)
Age at first sexual intercourse	< 18 years	51	71	1	
	> 18 years	92	192	0.667(0.431–1.033)	0.666(0.406–1.091)
Number of sexual partner	1	77	195	1	
	> 2	66	68	2.458(1.600–3.776)	2.112(1.297–3.441)
Number of husband’s sexual partner	1	32	103	1	
	> 2	111	160	2.233(1.403–3.554)	1.425(0.843–2.407)

## Discussion

The study aimed to identify prevalence and associated factors of Hr HPV among WLWH attending ART clinic in Shashemene town public health facilities. In this study the prevalence of Hr HPV among WLWH was 173 (35.2%), 95% CI (30.5%—40.1%). The identified prevalence of Hr HPV in this study was close with study conducted in Amazons which is 31.1% (95% CI: 25.8%-36.4%) of HIV positive women infected with Hr HPV [18]. The prevalence is lower than meta-analysis conducted in Kenya, Latin America and Caribbean whose magnitude were 64% and 51% respectively [26, 32]. The variation of the result may be due to different study design and study population. And also lower than cross sectional study conducted in public health institutions of the Kweneng East District Botswana, Cameroon and Bahamas whose magnitude were 40.4%, 46.43% and 78% respectively [33–35]. The variation between the findings may be attributable to geographical difference. On the other hand, difference in socio-economic, study area, methodology and study period could be a reason for variations.

Having history of STI, Endline viral load ≥ 50 copies/ml, Endline CD4 count < 200 cells/mm3 and having more than one life time sexual partner were identified as the predictors of Hr HPV infections.

In this study, WLWH who had a history of STI were significantly associated with Hr HPV infections which is 3-times more than those WLWH who did not have STI infections and it is consistent with study conducted in Brazil [36]. The possible explanation of this can be typically due to HPV infection being one type of STI and having others type of STI is crucial for entry and persistence of HPV through chronic cervical inflammation and ulceration in the cervical epithelium as well as through a reduction in host cell-mediated immunity caused by the STI, which may lead to decreased ability of spontaneous clearance and helps for persistence infections of HPV.

This study revealed that WLWH whose end line CD4 count < 200 cells/mm3 were strongly associated with Hr HPV infections than those whose End line CD4 count were ≥ 200 cells/mm3. This is line with previous study finding in the amazons, Brazil and Cameroon [18, 33]. The possible explanation for this might be due to the reason that having lower immunity status, as evidenced by a lower CD4 count, helps persistent infections of HPV by preventing spontaneous clearance of HPV.

This study also revealed that WLWH who had more than one-lifetime sexual partner were about two times more likely to be infected with Hr HPV than those WLWH who had an only one-lifetime sexual partner. This finding is in line with a similar study conducted in the rural Eastern Cape of South Africa, Denmark, and Brazil [20, 23, 37]. The possible explanation for this is that there are more than 200 different genotypes of HPV, which spread from one person to another by sexual contact, increased risk of getting Hr HPV when there is sexual contact with multiple sexual partners.

Further more WLWH whose endline HIV viral ≥ 50 copies/ml were about 3 times more likely to be infected with Hr HPV than those WLWH whose HIV vial load is < 50 copies/ml. This finding is line with previous study finding conducted in Bahamas [35]. This may be due to viral-viral interaction and/or inflammatory responses of HIV may interfere with a woman’s ability to support an effective immune response to HPV infection.

### Strength of study

Several significant strengths were present in this study. To my knowledge, it's the first study to measure Hr HPV prevalence among WLWH throughout the nation, which can be useful for other researchers. Availability of serology and genotyping in the study area. The collected data were both primary and secondary which keeps the quality of the data.

### Limitations of study

The study design (cross-sectional) not explain any causal relationship between any of the factors involved in the study. Some variables were collected from records and it was difficult to as required. Social desirable bias (some of the variables is about sexual history and drug abuse). Recall bias (Some women may have difficulty of remembering the exact time their age at first sexual intercourse).

## Conclusion

The results of this study indicate that there is a high prevalence of Hr HPV infection among WLWH in Shashemene town. A WLWH who have a lower end-line CD4 count, a high viral load, a history of STI, and/or more than one lifetime sexual partner were more likely to develop Hr HPV infection. These findings suggest that Hr HPV testing for cervical cancer screening in HIV-infected women would be necessary in Shashemene town, particularly for women who have identified factor.

### Recommendation

Shashemene town health office and health bureau should have to strengthen ART drug availability and early initiation, follow-up adherence, increase access to healthcare facilities. To healthcare providers and health extension workers, have to provide health education on the proper use of ART drugs and advice eating balanced diet, encourage correct and consistent use of condom and advice to limit number of sexual partner and in collaboration with community religious leaders and community leader need to mobilize and educate the health risk of having multiple sexual partners on the women and her family. Women living with HIV/AIDS encouraged to use ART drug properly, limit their life time sexual partner, consistent and proper use condoms, make regular screening of cervical cancer. Further researcher should be conducted using strong methodological approach to identify the underlying cause of Hr HPV.

## Data Availability

The datasets used generated and/or analyzed during the current study are not publicly available due to confidentiality but are available from corresponding authors upon request.

## Abbreviations

AID: Acquired Immune Deficiency Syndrome
ART: Anti-Retroviral Therapy
CC: Cervical Cancer
CDC: Communicable Disease Control
CGS: Council of Graduate Studies
DGC: Department Graduate Committee
FIGO: Federation of International Gynecology and Obstetrics
FMOH: Federal Ministry of Health
HIV: Human Immune Deficiency Virus
HPV: Human Papillomavirus
Hr HPV: High Risk Human Papillomavirus
ICC: Invasive Cervical Cancer
LMICs: Low and Middle Income Countries
LR: Low Risk
PCR: Polymerase Chain Reaction
RH: Reproductive Health
SGC: School Graduate Committee
SPSS: Statistical Package for Social Science
SSA: Sub Saharan Africa
STI: Sexually Transmitted Infections
VIA: Visual Inspection with Acetic Acid
WHO: World Health Organization
